# Rivaroxaban Treatment in Pediatric Patients With Hematologic Malignancies and Venous Thromboembolism: A Report of Four Cases

**DOI:** 10.7759/cureus.83487

**Published:** 2025-05-05

**Authors:** Ryohei Fukunaga, Toshikazu Itabashi, Koichi Kobayashi, Yujiro Tanabe, Jun Hayakawa, Takahiro Ueda

**Affiliations:** 1 Pediatrics, Nippon Medical School Hospital, Bunkyo, JPN; 2 Pediatric Medicine, Nippon Medical School Hospital, Bunkyo, JPN; 3 Pediatric Medicine, Nippon Medical School Musashi Kosugi Hospital, Kawasaki, JPN

**Keywords:** anticoagulants, direct oral anticoagulants, pediatric hematologic malignancy, rivaroxaban, thromboembolism

## Abstract

Venous thromboembolism is a serious complication in pediatric patients with hematologic malignancies, with acute pulmonary thromboembolism (PTE) posing a significant risk of cardiopulmonary collapse. Since the pediatric approval of rivaroxaban in 2021, marking the introduction of direct oral anticoagulants (DOACs) for this population, we have encountered four cases of thrombosis in children with hematologic malignancies. These included a six-year-old boy with B-cell precursor acute lymphoblastic leukemia treated with heparin followed by rivaroxaban for PTE; a 14-year-old girl with T-lymphoblastic lymphoma (T-LBL) who developed recurrent jugular vein thrombosis and discontinued rivaroxaban due to bleeding; a 10-year-old boy with T-LBL who survived life-threatening PTE with heparin and subsequent rivaroxaban therapy; and a 15-year-old girl with Hodgkin lymphoma who was managed with heparin and rivaroxaban for jugular vein thrombosis. All patients initially received heparin, followed by rivaroxaban for maintenance anticoagulation. Follow-up imaging confirmed thrombus resolution and no recurrence in all cases. Rivaroxaban proved effective in treating venous thrombosis in these pediatric patients, with successful outcomes and an overall favorable safety profile, underscoring the emerging role of DOACs in pediatric thrombosis management.

## Introduction

Venous thromboembolism (VTE), which includes pulmonary thromboembolism (PTE) and deep vein thrombosis, remains a serious clinical concern [1]. The in-hospital mortality rate for PTE is approximately 14%, increasing to 30% when complicated by heart failure. Notably, over 40% of deaths occur due to sudden cardiac arrest within one hour of symptom onset [2,3].

The primary risk factors for VTE are venous stasis, vascular endothelial injury, and hypercoagulability [1]. Malignancy is a well-established acquired risk factor due to its association with heightened coagulation activity [4,5]. Thrombosis may also worsen the clinical course of pediatric hematologic disorders [6-9]. In children with hematologic malignancies, several factors contribute to the elevated thrombotic risk, including chemotherapy-induced endothelial injury, tumor-related procoagulant activity, and the frequent use of central venous catheters. Additional contributors such as immobilization, infections, and corticosteroid use can further aggravate the hypercoagulable state [5,7].

Recent large-scale studies have reported that hospital-acquired VTE occurs in approximately one to three per 1,000 hospitalized children [8], and pediatric cancer patients are up to 30 times more likely to develop thrombosis than their healthy peers [7].

Traditionally, thrombosis has been managed with warfarin or heparin. However, the advent of direct oral anticoagulants (DOACs) has revolutionized treatment strategies [4,10,11]. The EINSTEIN-Jr trial demonstrated that rivaroxaban, when administered using weight-adjusted dosing in pediatric patients with acute VTE, showed comparable efficacy and safety to conventional heparin-based therapy, without an increase in adverse events [11]. Based on these findings, rivaroxaban received pediatric approval from the US FDA, the European Medicines Agency, and Japan’s regulatory authorities in 2021, signifying a global shift in pediatric thrombosis management.

Although there is a growing body of literature on the use of DOACs in children [12], reports specifically focusing on their use in pediatric hematologic malignancies remain limited [13]. Since 2021, our institution has treated four cases of thrombosis in pediatric patients with hematologic disorders using rivaroxaban. While one patient discontinued treatment due to a non-life-threatening bleeding event, no major bleeding complications were observed. Overall, the risk-benefit profile of rivaroxaban in these cases was deemed acceptable. These experiences contribute to the emerging evidence supporting the safety and efficacy of DOACs, particularly rivaroxaban, in pediatric patients with underlying hematologic conditions.

## Case presentation

Case 1: Six-year-old boy, B-cell precursor acute lymphoblastic leukemia (BCP-ALL)

A six-year-old boy was diagnosed with BCP-ALL and was treated using the ALL B-12 protocol (high risk) [14] in our department. Seven months after the initiation of treatment, during the first remission induction therapy, a drop in oxygen saturation (SpO₂ 80% on room air) was observed following a platelet transfusion, prompting further evaluation. Blood tests revealed a D-dimer level of 1.2 μg/mL, AT3 of 125.7%, and normal protein C and protein S levels (Table 1). Although the desaturation was initially suspected to be related to the platelet transfusion, the episode lacked clinical features typically associated with a transfusion reaction.

**Table 1:** Investigation results of Case 1 APTT, activated partial thromboplastin time; FDP, fibrin degradation product; FIB, fibrinogen; PT, prothrombin time

Laboratory test	Reference range	Test result
Hematology		
White blood cell count	4.0-10.5 × 10³/µL	0.8
Red Blood Cell Count	3.93-5.22 × 10⁶/µL	2.54
Hemoglobin	11.2-15.7 g/dL	7.7
Hematocrit	34.1-44.9%	23.3
Platelet count	150-400 × 10³/µL	33
Coagulation and fibrinolysis tests		
PT %	80.0-120.0%	106.8
APTT	24.0-39.0 seconds	31
FIB	170-400 mg/dL	618
D-dimer	<0.5 μg/mL	1.2
FDP	<10.0 μg/mL	-
Antithrombin III	80-120%	125.7
Protein C	64-146%	103
Protein S	64-149%	93.7

**Figure 1:**
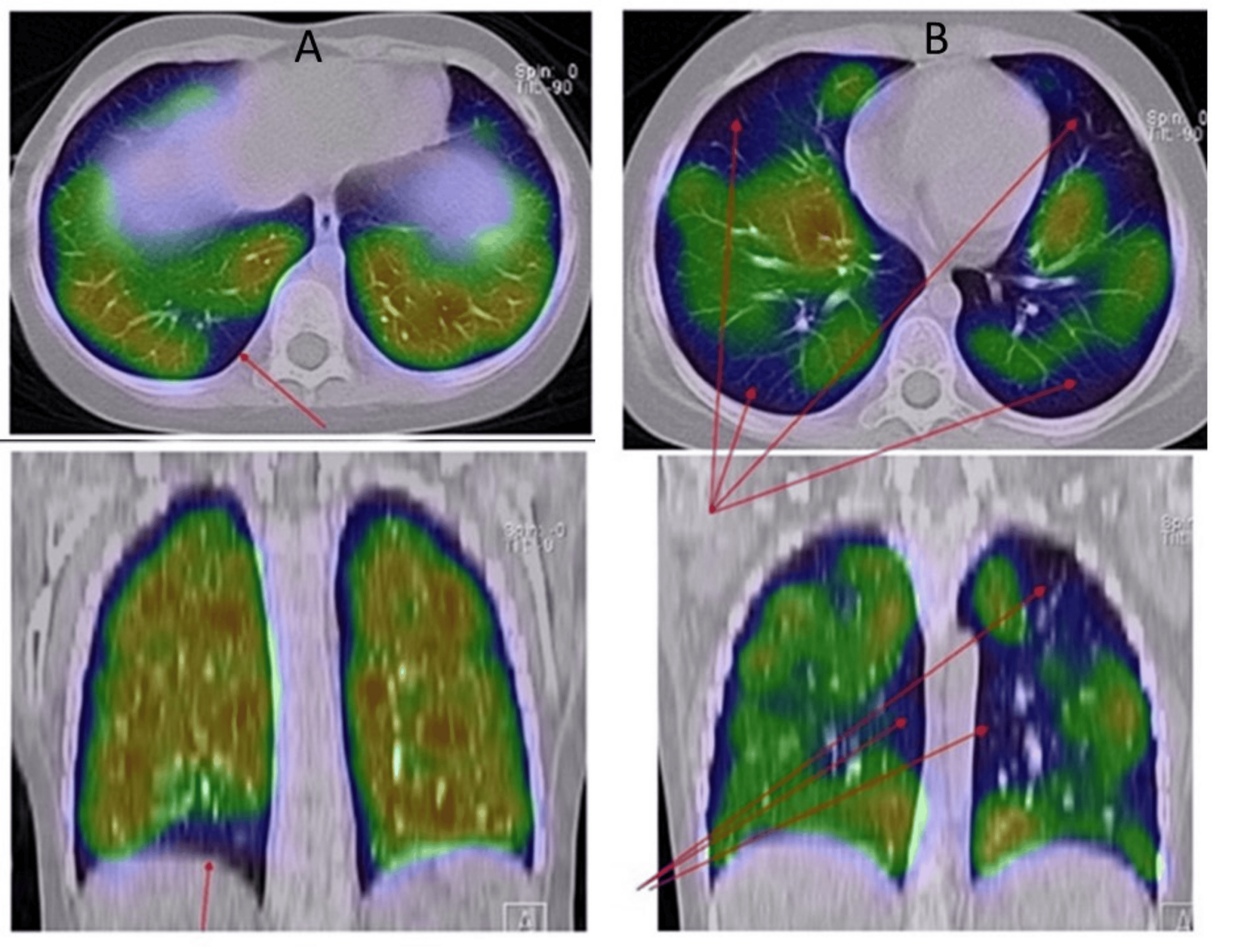
Pulmonary ventilation-perfusion scintigraphy of Case 1: (A) first episode and (B) second episode

A contrast-enhanced CT scan from the lower extremities to the lungs, conducted immediately after the onset of decreased oxygenation, revealed no evidence of a gross thrombus. However, a pulmonary ventilation-perfusion scintigraphy performed nine days later showed reduced accumulation in the S9 branch of the right lung base (Figure 1A), leading to a diagnosis of PTE. The patient remained asymptomatic, aside from occasional transient drops in peripheral oxygen saturation to approximately 90%, with no reported chest pain or dyspnea.

Following the diagnosis, low-molecular-weight heparin was administered during the high-risk period for thrombosis associated with L-asparaginase (L-Asp) therapy in the second re-induction phase. The patient progressed without further decreases in oxygen saturation or new signs of thrombosis.

During the third re-induction therapy - seven months after the initial PTE - the patient experienced another episode of nocturnal oxygen desaturation. Repeat contrast-enhanced CT scans from the lower extremities to the lungs again showed no thrombus. However, pulmonary ventilation-perfusion scintigraphy revealed reduced pulmonary blood flow in wedge-shaped areas in both lung fields, indicating exacerbation of PTE (Figure 1B). Blood tests showed a D-dimer level of 0.9 μg/mL and AT3 of 115.9%. Consequently, anticoagulation was switched from low-molecular-weight heparin to unfractionated heparin, and coagulation parameters were closely monitored.

Approximately two months after initiating unfractionated heparin for the second thrombotic episode, follow-up pulmonary ventilation-perfusion scintigraphy confirmed thrombus resolution. This imaging was performed at the end of inpatient chemotherapy, once the patient had completed the acute treatment phase and was clinically stable. Despite radiologic resolution, oral rivaroxaban (10 mg/day) was initiated at the start of maintenance therapy to minimize recurrence risk in the setting of ongoing malignancy-related hypercoagulability. Rivaroxaban was selected due to its once-daily oral dosing, favorable pharmacokinetics, and minimal monitoring requirements - benefits particularly suited to outpatient pediatric care.

After approximately three months of rivaroxaban therapy, repeat pulmonary ventilation-perfusion scintigraphy confirmed complete thrombus resolution, and rivaroxaban was discontinued. The patient has remained free of thromboembolic recurrence since the completion of therapy.

Case 2: 14-year-old girl, T-lymphoblastic lymphoma (T-LBL)

One month prior to admission, the patient developed progressive swelling in her neck. She visited her previous clinic, where a mediastinal mass was identified. She was subsequently referred to our department for further evaluation and treatment.

A contrast-enhanced CT scan of the chest revealed an 80 mm soft tissue mass in the left upper mediastinum, causing rightward tracheal deviation due to compression. Given the risk of asphyxia from the mass, steroid therapy was initiated prior to biopsy. Five days after starting steroid treatment, a biopsy was performed, and the patient was diagnosed with T-LBL, stage III. Treatment was initiated using the NHL-BFM95 protocol [15].

**Figure 2:**
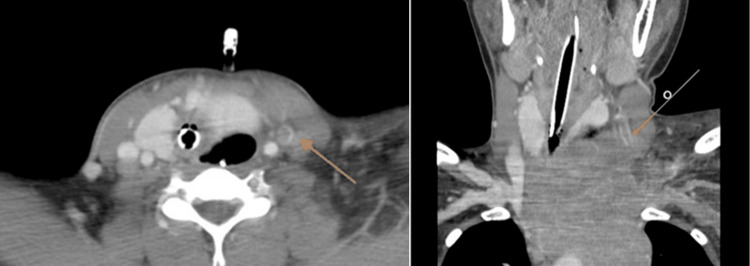
Thrombus in the left jugular vein of Case 2

On day 6 after the initiation of treatment, a follow-up contrast-enhanced CT scan to assess treatment response revealed a thrombus in the left internal jugular vein (Figure 2). In this case, the use of IV steroids and physical compression of the vein by the tumor were considered contributing risk factors for thrombosis. Consequently, anticoagulation therapy with unfractionated heparin was started. Blood tests showed a D-dimer level of 3.0 μg/mL, with normal protein C and protein S levels (Table 2).

**Table 2:** Investigation results of Case 2 APTT, activated partial thromboplastin time; FDP, fibrin degradation product; FIB, fibrinogen; PT, prothrombin time

Laboratory test	Reference range	Test result
Hematology		
White blood cell count	4.0-10.5 × 10³/µL	6.4
Red blood cell count	3.93-5.22 × 10⁶/µL	4.41
Hemoglobin	11.2-15.7 g/dL	13.1
Hematocrit	34.1-44.9%	39.7
Platelet count	150-400 × 10³/µL	188
Coagulation and fibrinolysis tests		
PT %	80.0-120.0%	77.1
APTT	24.0-39.0 seconds	25.9
FIB	170-400 mg/dL	274
D-dimer	<0.5 μg/mL	3
FDP	<10.0 μg/mL	-
Antithrombin III	80-120%	125.9
Protein C	64-146%	98
Protein S	64-149%	74.3

Three months after initiating heparin therapy, contrast-enhanced CT confirmed that the thrombus had resolved, and heparin was subsequently discontinued. The patient was initially monitored without anticoagulants while the underlying disease was managed. A central venous catheter was inserted, and approximately two weeks later, a neck vein ultrasound revealed the formation of a new thrombus. At that time, blood tests showed a normal D-dimer level of 0.7 μg/mL. Oral rivaroxaban therapy (15 mg/day) was initiated. The decision to use a DOAC instead of heparin was based on the recent approval of DOACs for pediatric use in Japan and the preference for a more convenient oral treatment that could be managed at home, reducing the need for hospital visits and injections.

After two months of treatment, the thrombus had completely resolved. However, three months after starting rivaroxaban, the medication was discontinued due to side effects, including epistaxis and hematuria. At the time of the bleeding event, prothrombin time, activated partial thromboplastin time (APTT), and D-dimer levels were all within normal ranges, consistent with findings in other cases. This suggested that the bleeding was not associated with significant coagulation abnormalities. Although the bleeding was not life-threatening, rivaroxaban was discontinued for safety reasons. The symptoms, which emerged three months after initiating DOAC therapy, resolved upon discontinuation of the medication. This delayed onset of side effects is less common, as most reports describe bleeding occurring soon after treatment initiation. After stopping rivaroxaban, no thrombus recurrence was observed. The patient has since completed maintenance therapy and remains in remission.

Case 3: 10-year-old boy, T-LBL

Two weeks prior to admission, the patient developed a fever, swelling in the left side of the neck, and pain in the right axilla. Upon admission, a mass was detected in the left neck and mediastinum. His oxygen saturation (SpO₂) was 97% on room air. Blood tests revealed a D-dimer level of 3.9 μg/mL, with normal protein C and protein S levels (Table 3).

**Table 3:** Investigation results of Case 3 APTT, activated partial thromboplastin time; FDP, fibrin degradation product; FIB, fibrinogen; PT, prothrombin time

Laboratory test	Reference range	Test result
Hematology		
White blood cell count	4.0-10.5 × 10³/µL	12.5
Red blood cell count	3.93-5.22 × 10⁶/µL	4.59
Hemoglobin	11.2-15.7 g/dL	11.8
Hematocrit	34.1-44.9%	36.1
Platelet count	150-400 × 10³/µL	327
Coagulation and fibrinolysis tests		
PT %	80.0-120.0%	110
APTT	24.0-39.0 seconds	34
FIB	170-400 mg/dL	650
D-dimer	<0.5 μg/mL	3.9
FDP	<10.0 μg/mL	11.7
Antithrombin III	80-120%	108.9
Protein C	64-146%	116
Protein S	64-149%	124

A contrast-enhanced CT scan of the neck revealed a 100 × 90 mm mass with heterogeneous enhancement in the anterior mediastinum, rightward tracheal deviation, enlarged left supraclavicular lymph nodes, and a left pleural effusion. Neck ultrasonography confirmed a thrombus in the left internal jugular vein.

Due to the patient’s unstable condition, particularly respiratory compromise, a biopsy was not performed. Instead, T-LBL was diagnosed based on pleural fluid cytology, with flow cytometry further supporting the diagnosis. Prednisolone monotherapy was initiated along with anticoagulation using low-molecular-weight heparin.

**Figure 3:**
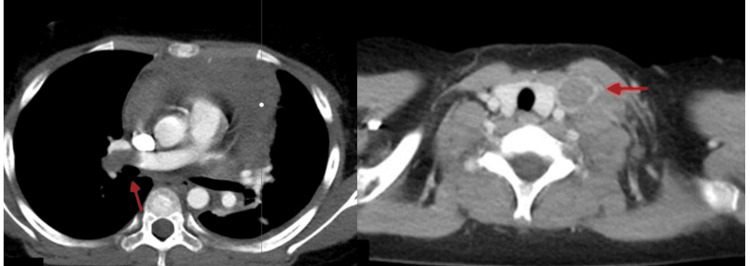
Thrombus in the main trunk of the right pulmonary artery and left internal jugular vein of Case 3

On day 4 of therapy, the patient experienced sudden oxygen desaturation and dyspnea. A contrast-enhanced CT scan revealed thrombi in the main trunk of the right pulmonary artery and in the left internal jugular vein (Figure 3). Shortly after imaging, the patient went into cardiac arrest in the hospital room. Cardiopulmonary resuscitation was initiated, and he was transferred to the emergency room, where spontaneous circulation was restored after approximately eight minutes.

Unfractionated heparin was administered in the intensive care unit. Nine days later, after his condition had stabilized, anticoagulation was switched to rivaroxaban (15 mg/day). After three months of therapy, a repeat contrast-enhanced CT scan confirmed that the thrombus had resolved. Rivaroxaban was continued for a total of nine months, until the completion of hospitalization and removal of the central venous catheter. The patient has since completed maintenance therapy and remains in remission.

Case 4: 15-year-old girl, Hodgkin lymphoma (HL)

The patient was referred to our department for further assessment and care after her primary care physician inadvertently discovered a cervical mass. A contrast-enhanced CT scan upon admission revealed enlarged lymph nodes and thymus from the left cervical to supraclavicular regions. The tumor was found to be significantly compressing the left internal carotid artery, raising concerns about a high risk of thrombosis. As a precaution, unfractionated heparin therapy was initiated.

Subsequent neck ultrasonography revealed a thrombus in the left internal jugular vein. The intensity of unfractionated heparin therapy was increased and maintained. Blood tests showed a D-dimer level of 0.8 μg/mL, with normal protein C and protein S levels (Table 4).

**Table 4:** Investigation results of Case 4 APTT, activated partial thromboplastin time; FDP, fibrin degradation product; FIB, fibrinogen; PT, prothrombin time

Laboratory test	Reference range	Test result
Hematology		
White blood cell count	4.0-10.5 × 10³/µL	7.5
Red blood cell count	3.93-5.22 × 10⁶/µL	4.97
Hemoglobin	11.2-15.7 g/dL	13.6
Hematocrit	34.1-44.9%	41.7
Platelet count	150-400 × 10³/µL	278
Coagulation and fibrinolysis tests		
PT %	80.0-120.0%	95.1
APTT	24.0-39.0 sec	31.9
FIB	170-400 mg/dL	407
D-dimer	<0.5 μg/mL	0.8
FDP	<10.0 μg/mL	-
Antithrombin III	80-120%	111
Protein C	64-146%	101
Protein S	64-149%	92

Following a biopsy of the cervical lymph node, echocardiography revealed that the thrombus in the jugular vein had resolved. The possibility of PTE due to thrombus migration via physical decompression was considered, but contrast-enhanced CT revealed no evidence of thrombosis. The biopsy results confirmed the diagnosis of HL. She was treated according to the HL-14(IR) protocol [16].

On day 13, after the initiation of heparin therapy, the patient was switched to rivaroxaban (15 mg/day) and continued treatment following the HL-14 intermediate-risk protocol [16]. After six months of rivaroxaban therapy, contrast-enhanced CT revealed no evidence of thrombus, and anticoagulation therapy was discontinued. The patient remains in remission.

A summary of the four cases is presented in Table 5.

**Table 5:** Summary of the four cases BCP-ALL, B-cell precursor acute lymphoblastic leukemia; HL, Hodgkin lymphoma; L-Asp, L-asparaginase; T-LBL, T-lymphoblastic lymphoma

Case	Age	Gender	Underlying disease (stage)	Treatment protocol referenced	Treatment block at the diagnosis of thrombosis	Location of the thrombus	Risk factors for thrombosis	Diagnostic method	Treatment of thrombosis	Side effects of treatment	Duration until thrombus disappearance
Case 1	6	M	BCP-ALL (high risk)	ALL-B12 (14)	1st III+L, 3rd III+L	Right pulmonary artery	Long-term indwelling catheter, L-Asp	Pulmonary ventilation-perfusion scintigraphy	Heparin → rivaroxaban	None	Eight months
Case 2	14	F	T-LBL (stage III)	NHL-BFM95 (15)	Induction, protocol M	Left internal jugular vein	Venous compression, steroids	Contrast-enhanced CT	Heparin → rivaroxaban	Epistaxis, hematuria	Three months
Case 3	10	M	T-LBL (stage III)	NHL-BFM95 (15)	Before treatment initiation	Main trunk of the right pulmonary artery and left internal jugular vein	Venous compression	Neck ultrasound	Heparin → rivaroxaban	None	Three months
Case 4	15	F	HL (stage II EA)	HL-14 (16)	Before treatment initiation	Left internal jugular vein	Venous compression	Neck ultrasound	Heparin → rivaroxaban	None	Two days

## Discussion

The mortality rate of acute PTE can reach up to 30% in undiagnosed or untreated cases. However, early diagnosis and appropriate treatment significantly improve survival rates [2]. Sudden symptoms such as dyspnea, chest pain, tachycardia, syncope, wheezing, or a cold sensation should raise clinical suspicion for PTE and prompt immediate diagnostic evaluation. Despite this, PTE remains challenging to diagnose, particularly because it can arise abruptly from an otherwise asymptomatic state [1]. In pediatric patients, this difficulty is compounded by the tendency to present with fewer or less specific symptoms compared to adults - a factor that must be carefully considered in the context of malignancy treatment [2].

Thrombosis can be evaluated using several diagnostic modalities, including chest X-rays, electrocardiograms, D-dimer tests, contrast-enhanced CT, pulmonary ventilation-perfusion (V/Q) scintigraphy, and deep vein ultrasonography [17]. However, no single method is definitive. Elevated D-dimer levels can occur in malignancy even in the absence of thrombus formation, so this marker alone should not be used for diagnosis. Among the four cases presented, markedly elevated D-dimer was observed only in Case 3, where pulmonary artery thromboembolism led to cardiac arrest. In other cases, such as Case 1, contrast-enhanced CT failed to detect thrombosis, underscoring the utility of pulmonary V/Q scintigraphy when CT is inconclusive. Small or subsegmental pulmonary emboli may be missed on CT, particularly in pediatric patients due to motion artifacts or technical limitations. In such situations, V/Q scintigraphy remains a valuable diagnostic alternative. Typically, CT pulmonary angiography is the initial imaging modality for suspected pulmonary embolism in pediatric oncology patients, while V/Q scintigraphy is used for confirmation, especially in detecting small or subsegmental emboli.

In pediatric oncology, thrombosis is often multifactorial - resulting from tumor-related hypercoagulability, drug-induced prothrombotic effects, use of central venous catheters, surgical interventions, radiation therapy, or thrombotic complications [5,18]. Among antineoplastic agents, corticosteroids and L-Asp are particularly known for their thrombogenic potential [5]. Furthermore, malignancies involving longitudinal lesions may cause physical compression of the vena cava, increasing the risk of thrombosis and asphyxia, as observed in Cases 2 to 4. Therefore, clinicians must remain vigilant. Thrombus control is especially critical during the induction phase of acute lymphoblastic leukemia treatment, where prolonged steroid use can exacerbate hypercoagulability.

DOACs, such as rivaroxaban, function by selectively inhibiting Factor Xa, effectively preventing thrombin generation and subsequent thrombus formation. Compared to traditional anticoagulants, DOACs offer advantages such as fewer drug interactions and reduced need for routine laboratory monitoring, making them well-suited to the complex pharmacological landscape of modern cancer treatment [19].

In our four cases, DOAC treatment led to complete thrombus resolution. In two of the cases, we also believe thromboprophylaxis was achieved. These findings align with previous reports supporting the prophylactic and therapeutic potential of rivaroxaban in cancer-associated thrombosis [20]. Oral administration of DOACs may thus be a promising strategy not only for treating thrombosis but also for preventing recurrence in pediatric patients with hematologic malignancies. According to the EINSTEIN-Jr trial, 10 out of 329 pediatric patients treated with rivaroxaban experienced bleeding events, including one case of life-threatening major bleeding. Minor bleeding events included gastric mucosal bleeding (n = 4), urinary occult blood (n = 2), skin bleeding (n = 1), and nasal/oral bleeding (n = 3) [11]. Among our patients, one experienced minor bleeding, but no major adverse effects were observed. While the incidence of minor bleeding in our series (1/4, or 25%) appears higher than the approximately 3% reported in the EINSTEIN-Jr trial, this may reflect the elevated bleeding risk in pediatric oncology patients, who are often subject to additional bleeding risks from chemotherapy, tumor-associated coagulopathies, and invasive procedures.

## Conclusions

Our findings contribute to the growing body of evidence supporting the efficacy and safety of DOACs in pediatric thrombosis management. In particular, rivaroxaban appears to be a promising option for both treatment and prevention of thrombotic events in children with hematologic malignancies. However, further large-scale studies are needed to better define its optimal use and assess long-term safety in this unique patient population.
